# News and Events of the Week

**Published:** 1910-04-30

**Authors:** 


### News and Events of the Week.

The first annual dinner of the University of London Officers' Training Corps will be held at the Victoria Restaurant, Victoria Station, on Wednesday, May 18, 1910, at 7.30 p.m., and will be followed by a smoking concert. The corps will be on parade in the afternoon at the University.

A coroner's physician in New York has been made the defendant in a suit for £5,000. The plaintiff alleges that the defendant illegally abstracted and retained her son's spleen and heart when he made an autopsy on the young man, who came to his death as the result of an accident which ruptured his spleen.

Mr. F. A. Southam, who succeeded Mr. Walter Whitehead as Professor of Clinical Surgery at Manchester University ten years ago, has resigned his professorship, and Mr. William Thorburn, of Manchester, has been appointed to the vacancy. Mr. Thorburn is an examiner of the University of London, and has contributed largely to surgical literature.

At a recent meeting at Hounslow a motion to establish a consumption sanatorium for Middlesex was carried, and a provisional committee formed. Sir Douglas Powell said that sanatoria were the only means of curing consumption, and to cope with the disease in England 20,000 beds, costing £250,000, were needed. Maintenance and treatment would cost annually another £250,000. He thought that sum could be raised if the thousands of pounds at present frittered away by friendly societies in attempting to treat the disease were co-ordinated.

At an examination in sanitary science as applied to buildings and public works, held at Blackburn on April 8 and 9, 1910, by the Royal Sanitary Institute, the following candidate presented himself and was granted a certificate: Maybury Sydney Allan Lewis, of Crewe. At an examination for women health visitors and school nurses, held at Blackburn on April 8 and 9, 1910, five candidates presented themselves and were granted certificates. At an examination for inspectors of nuisances, held at Blackburn on April 8 and 9, 1910, twenty-six candidates presented themselves, of whom eleven were certified, as regards their sanitary knowledge, competent to discharge the duties of inspector of nuisances under the Public Health Act, 1875.

At the annual general meeting of the National Association for the Establishment and Maintenance of Sanatoria for workers suffering from tuberculosis, of which Princess Christian is President, the report for 1909 was read. It showed that 281 patients were treated on the most modern lines at the National Sanatorium, Benenden, Kent, and that of the patients who have left the sanatorium with the disease arrested, 81.4 per cent. are still doing full work. Sixty-three beds were maintained there by friendly societies and workers' organisations, which subscribed £65 per bed per annum, an increase on the previous year. Necessary extension of buildings has involved considerable expense, £8,000 of which still remains unpaid. Subscriptions and donations to the capital expenditure will be gratefully acknowledged by the Secretary, National Sanatorium Association, 52 Gray's Inn Road, London, W.C., or National Sanatorium, Benenden, Kent.

Professor Sir William Whitla, M.D., LL.D., has been appointed to represent the Queen's University of Belfast on the General Medical Council for three years.

Mr. Walter Runciman, President of the Board of Education, has appointed a Departmental Committee to consider and report upon the functions and constitution of the Royal College of Art and its relations to the Schools of Art in London and throughout the country.

The weekly return of births and deaths in London and in 76 other great towns, issued by authority of the Registrar-General, states that the deaths registered last week in 77 great towns of England and Wales corresponded to an annual rate of 13.7 per 1,000 of their aggregate population, estimated at 16,940,895 persons in the middle of this year. No death from small-pox was registered in any of the 77 towns. In London 2,334 births and 1,226 deaths were registered. Allowing for increase of population the births were 86 and the deaths 268 below the average numbers in the corresponding weeks of the previous five years. The annual death-rate from all causes, which had been 14.5, 14.7, and 14.8 per 1,000 in the preceding three weeks, fell last week to 13.1.

A reception was held on Friday afternoon by Muriel, Viscountess Helmsley, at the Ideal Home Exhibition, Olympia, for the National Society of Day Nurseries. The Society is exhibiting a model *crèche*, and Lady Helmsley has personally supervised all the arrangements, and is in constant attendance to explain details to those interested in day nurseries. The greatest interest was shown by those present in this instruction, and this Society's *crèche* has been thronged by the general public during the past week. It is clear that the work of the Society in helping to establish and improve day nurseries all over the country is meeting a genuine need. Donations and annual subscriptions will be gratefully received by the acting treasurer, Mr. Hoffnung-Goldsmid, or by the secretary, Mr. Herbert H. Jennings, at the Society's Offices, 1 Sydney Street, Fulham Road, S.W.

The Westminster City Council have decided to place a contract with the Gas Light and Coke Company for the lighting of Regent Street, Piccadilly Circus, St. James's Street, and Pall Mall by the latest form of high-pressure gas lamp in place of the existing electric arc lamps. Especial interest attaches to this decision from the fact that Pall Mall was the first thoroughfare in the world to be lighted by gas, the original lamps being installed over a hundred years ago. The inverted mantle has evidently given the centenarian light a new lease of life. It will be remembered that the screen in front of Buckingham Palace and the whole of Fleet Street are now lighted by high-pressure gas lamps. The decision of the Westminster Council (which also provides for the improved gaslighting of Victoria Street and many other thoroughfares in the City by inverted burners) was based upon the fact that the tenders received from the gas and electricity companies revealed a difference of from 30 to 80 per cent. in favour of gas, light for light—a matter of some importance for institutional authorities who do not possess their own electric plant.

